# An Attempt Likely to Fail

**Published:** 1898-03

**Authors:** 


An attempt on the part of President Canfield, of the Ohio State University to get Starling College and the Ohio Medical University to consolidate, and then become part of the State University, is likely to fail. The majority of the faculty of Medical University is opposed to the scheme of consolidation, and say it will never occur.

